# Multimorbidity and Disability Among Venezuelan Migrants: A Population-Based Survey in Peru

**DOI:** 10.1007/s10903-021-01259-8

**Published:** 2021-08-27

**Authors:** Antonio Bernabe-Ortiz, Rodrigo M. Carrillo-Larco

**Affiliations:** 1 CRONICAS Center of Excellence in Chronic Diseases, Universidad Peruana Cayetano Heredia, Av Armendariz 445, Miraflores, Lima, Peru; 2 School of Public Health and Administration, Universidad Peruana Cayetano Heredia, Lima, Peru; 3 Universidad Científica del Sur, Lima, Peru; 4 Department of Epidemiology and Biostatistics, School of Public Health, Imperial College London, London, UK

**Keywords:** Humanitarian crisis, Migration, Non-communicable diseases

## Abstract

The political and economic crisis in Venezuela has originated an unprecedented migration. As of November 2020, 1.04 million Venezuelans have moved to Peru. Understanding their health profile is needed to identify their needs, provide care and secure resources without affecting the healthcare of nationals. We quantified the burden of multimorbidity and disability in the Venezuelan population in Peru. We analyzed the 2018 Survey of Venezuelan Population Living in Peru; population-based with random sampling survey in six cities in Peru. Participants were asked about the presence of 12 chronic conditions (self-reported); this information was grouped into 0, 1 and ≥ 2 conditions (i.e., multimorbidity). Disability was also ascertained with a self-reported questionnaire adapted from the short version of the Washington Group on Disability Statistics. Socioeconomic variables were analyzed as potential determinants. Variables were described with frequencies and 95% confidence interval (95% CI), compared with Chi2 test, and association estimates were derived with a Poisson regression reporting prevalence ratio and 95% CI. Results accounted for the complex survey design. The analysis included 7554 migrants, mean age 31.8 (SD: 10.2), 46.6% were women, 31.7% migrated alone and 5.6% had refugee status. The prevalence of multimorbidity was 0.6% (95% CI 0.4–0.9%), and was often present in women (p < 0.001), people ≥ 50 years (p < 0.001) and those without recent job (p < 0.001). The prevalence of disability was 2.0% (95% CI 1.5–2.7%), and was common among people ≥ 50 years (p < 0.001) and those without recent job (p < 0.001). Migration alone and refugee status were not associated with multimorbidity or disability. The self-reported prevalence of multimorbidity and disability in Venezuelan migrants in Peru was low, and were not strongly influenced by migration status. While these results could suggest a healthy migrant effect, the healthcare system should be prepared to deliver acute and preventive care for these migrants, while also securing primary prevention to delay the onset of chronic conditions in this population.

## Introduction

Multimorbidity is defined as the presence of two or more chronic conditions in the same individual [1]. A systematic review reported a pooled multimorbidity prevalence of 33% using population-based studies, with varying results depending on socio-economic status and the proportion of elderly (≥ 60 years) people in the population [2]. In addition to this high prevalence, multimorbidity and its consequences are rising in low- and middle-income countries [3]. Disability, defined as a limitation in functioning at the body, person or societal level in one or more life domains [4, 5], can be a consequence of multimorbidity [6]. In addition, pre-existing disability may be also an exposure for future disability if this is not appropriately treated.

Migration may negatively affect health, but this effect is heterogeneous depending on key factors. For example, a recent systematic review, published in 2020, reported an association between age at migration and psychotic disorders among first-generation migrant groups [7]. Another systematic review from 2014 reported the relationship between international migration and the development of asthma and allergic diseases, highlighting the impact of environment on health [8]. Finally, within-country migration has also been considered a potential factor involved in differential effect on cardiovascular health [9]. Nevertheless, forced migration because of conflicts or disasters usually has a negative impact on morbidity patterns, conditioned on the individual’s health before migration, socio-economic and environmental profiles, disease patterns, cultural norms and practices, and access to healthcare at destination [10]. The political crisis in Venezuela has led to the departure of 5.45 million people since 2014, and approximately 1.04 (19%) million are estimated to be in Peru as of November 2020 [11].

The health vulnerability of Venezuelan migrants may be an issue of concern as the circumstances of migration are not purely economic, resembling those of forced migration. Moreover, because the massive migration, health disparities and health access may be relevant issues to be considered, in addition to the burden of the Peruvian healthcare system. The Peruvian National Institute of Statistics and Informatics (INEI in Spanish) conducted the Survey of Venezuelan Population Living in Peru (ENPOVE in Spanish) in December 2018, when the number of Venezuelan migrants in Peru was estimated in 635,000 [12]. This survey contains information regarding sociodemographic characteristics, vulnerabilities, health status, education, and employment conditions of Venezuelan migrants in Peru with the aim of designing and generating appropriately policies for this population.

Thus, using this information, we aimed to describe health, multimorbidity and disability patterns among Venezuelan migrants in Peru using the aforementioned survey. In so doing, we provide an overall picture of the health status of the Venezuelan population in Peru to inform national and international organization working to improve their health standards.

## Methods

### Study Design and Setting

The ENPOVE survey is a self-reported population-based study conducted in the capital city of six (out of 25) Peruvian regions: Arequipa, Callao, Cusco, Lima, Trujillo and Tumbes. These cities were selected because they host approximately 85% of the Venezuelan population in Peru [13]. The ENPOVE aimed at providing statistical information about the sociodemographic, employment, migration, and health status of Venezuelan migrants living in Peru.

### Sampling

The ENPOVE used a bietapic and stratified sampling strategy, independent for each city of the study. The survey was conducted by the INEI between November and December 2018 [14]. The primary sampling units were groups of blocks of households where Venezuelan population were living; whereas the second sampling unit was the household where at least an individual with Venezuelan nationality was found. In the selected households, the total number of Venezuelan individuals and their ages were recorded and then interviewed.

### Study Participants

The study population was comprised by Venezuelan migrants living in the urban area of the aforementioned cities [14]. Each member of the selected household, ≥ 12 years old, was interviewed using a face-to-face technique. For those aged < 12 years the information was provided by the household head. The survey approach, interview techniques and questionnaires used in this survey are available elsewhere [13]. We analyzed information of those aged ≥ 18 years and those with complete data in the variables of interest; we excluded pregnant women.

### Definition of Variables

There were two outcomes of interest. The first one was the number of chronic conditions selected from a list of 12 self-reported diseases: arthritis, hypertension, asthma, rheumatism, type 2 diabetes, tuberculosis, hypercholesterolemia, chronic pulmonary disease, cancer, mental health disorders, heart disease, and HIV/AIDS. These conditions were selected as they require long-term medical treatment and repeated clinical appointments with potential impact on the Peruvian health system. These conditions were grouped into none, one or two (i.e., multimorbidity [1]) chronic conditions. The second outcome of interest was disability, defined as any permanent limitation, if physical, mental, intellectual or sensorial. Questions regarding disability addressed whether the participants had any movement limitation, visual impairment, communication difficulty, hearing impairment, difficulty with learning and/or remembering, and difficulty with social relationships; this information was collected with six no/yes questions. Participants who responded “yes” to any of these questions were deemed to have a disability. These questions were adapted from the recommended short version of the Washington Group on Disability Statistics [15] and previously used for Peruvian national estimates [16].

We also analyzed other potential health determinants. The first one assessed whether the participant decided to migrate from Venezuela to Peru alone (no vs. yes); the second one evaluated whether the participant migrated as a refugee (self-reported). Socio-economic variables were also studied: sex (female vs. male); age (< 30, 30–39, 40–49, ≥ 50 years); education level (< 7, 7–11, and ≥ 12 years); socio-economic status based on a wealth index and split into low, middle and high; marital status (never married, currently married, and previously married); region (Arequipa, Callao, Cusco, Lima, Trujillo, and Tumbes); if the participant worked last week (no vs. yes); and having any health insurance (no vs. yes). Among those with at least a chronic condition, questions regarding access to appropriate treatment were also asked.

### Procedures

Data collection was conducted by trained staff using tablets [14]. The tablet application had a control assurance system to guarantee appropriate information quality. Specific handbooks were created for fieldwork activities, and a pilot was carried out to test tools and documents to be used in the survey [13].

### Statistical Analysis

Stata 16 for Windows (StataCorp, College Station, TX, US) was used for the analyses. All estimates were calculated taking into account sample strata, primary sampling units and population weights using SVY commands. Appropriate techniques were used for estimating results in subpopulations of interest [17].

Population characteristics were described according to sex of participants and comparisons were done using the Chi-squared test. Prevalence of multimorbidity and disability were estimated and reported with their 95% confidence intervals (95% CI). Sociodemographic variables were assessed as potential factors associated with the number of chronic conditions and disability. Finally, multivariable models, adjusted by sociodemographic variables, were built using Poisson regression models to assess the association between migration alone and refugee status with the outcomes of interest; prevalence ratio (PR) and 95% CI were reported.

### Ethics

The ENPOVE survey is a publicly available de-identified database stored at a national repository [18]; therefore, approval from an Institutional Review Board was not considered mandatory. The database used does not provide any information that would have breached participant’s confidentiality.

## Results

### Characteristics of the Study Population

A total of 9852 subjects were enrolled in the study, but 2091 (21.2%) were excluded: 1956 because they were aged < 18 years and 135 because of pregnancy; in addition, 207 (2.1%) records were not considered in the analyses because of missing observations. Therefore, information of 7554 migrants, mean age: 31.8 (SD: 10.2) years, and 3614 (46.6%) females, was analyzed. Of note, 2150 (31.7%) individuals reported having migrated alone compared to 68.3% who reported traveling in group (family or friends), and 745 (5.6%) reported being a refugee to obtain permission to entry to Peru. Projections yielded a total of 658,000 Venezuelan migrants living in the selected cities, with a subsample according to our selection criteria of 504,000 individuals.

When the characteristics of the study population was evaluated according to migrant’s sex, males tend to be younger (p < 0.001), single (p < 0.001), and have migrated alone (p < 0.001); whereas females were better educated (p < 0.001) and have not worked the week before the interview (p < 0.001; Table 1).

**Table 1:** Characteristics of the study population by sex

	Participant’s sex		p-value
	Females	Males	
	(n = 3479)	(n = 4075)	
Age (in years)			< 0.001
< 30	1776 (51.2%)	2082 (51.1%)	
30–39	929 (26.3%)	1321 (32.5%)	
40–49	450 (13.3%)	476 (11.5%)	
≥ 50	324 (9.2%)	196 (4.9%)	
Education level (in years)			< 0.001
< 7	326 (8.6%)	434 (10.9%)	
7–11	953 (27.2%)	1413 (34.2%)	
≥ 12	2200 (64.2%)	2228 (54.9%)	
Socioeconomic status			0.480
Low	325 (11.6%)	411 (12.0%)	
Middle	1536 (54.3%)	1841 (55.5%)	
High	1618 (34.1%)	1823 (32.5%)	
Marital status			< 0.001
Never married	1169 (34.1%)	1592 (40.5%)	
Currently married	2030 (58.5%)	2404 (58.0%)	
Previously married	280 (7.4%)	79 (1.5%)	
Region			0.470
Lima	1672 (48.1%)	1945 (47.7%)	
Arequipa	529 (15.2%)	589 (14.4%)	
Callao	398 (11.4%)	467 (11.5%)	
Cusco	219 (6.3%)	306 (7.5%)	
Trujillo	475 (13.6%)	581 (14.3%)	
Tumbes	186 (5.4%)	187 (4.6%)	
Migrated alone			< 0.001
Yes	733 (23.7%)	1393 (38.6%)	
Refugee			0.007
Yes	334 (6.8%)	395 (4.6%)	
Worked last week			< 0.001
No	824 (25.2%)	285 (7.2%)	
Health insurance			0.410
Yes	187 (5.0%)	219 (5.5%)	

Percentages are in columns

### Chronic Conditions, Multimorbidity and Disability

Out of the 7554 migrants, 808 (11.0%; 95% CI 9.8–12.3%) reported having only one of the chronic conditions evaluated, whereas 47 (0.6%; 95% CI 0.4–0.9%) reported having ≥ 2 chronic conditions. Asthma (3.4%) was the most frequent chronic condition reported, followed by hypertension (2.5%) and type 2 diabetes (0.8%). Multimorbidity was more common among females (p < 0.001), those aged ≥ 50 years (p < 0.001), those previously married (p < 0.001), and those who did not work last week (p < 0.001; Table 2). Among those with at least one chronic condition (n = 865), only 103 (10.9%) reported receiving treatment, whereas 93 (11.3%) reported receiving some therapies but not in a frequent way, and 669 (77.8%) did not received any treatment for their conditions.

**Table 2:** Characteristics of the study population by multimorbidity profile

	Number of chronic conditions			p-value
	0	1	≥ 2	
	(n = 6699)	(n = 808)	(n = 47)	
Sex				< 0.001
Female	2960 (85.7%)	480 (13.2%)	39 (1.1%)	
Male	3739 (90.8%)	328 (9.0%)	8 (0.2%)	
Age (in years)				< 0.001
< 30	3529 (91.4%)	324 (8.5%)	5 (0.1%)	
30–39	2024 (89.3%)	215 (9.9%)	11 (0.8%)	
40–49	802 (84.7%)	119 (15.0%)	5 (0.3%)	
≥ 50	344 (69.2%)	150 (26.8%)	26 (4.0%)	
Education level (in years)				0.99
< 7	660 (88.3%)	91 (11.1%)	9 (0.6%)	
7–11	2127 (88.3%)	226 (11.1%)	13 (0.6%)	
≥ 12	3912 (88.5%)	491 (10.9%)	25 (0.6%)	
Socioeconomic status				0.57
Low	665 (89.4%)	64 (9.7%)	7 (0.9%)	
Middle	3007 (88.6%)	348 (10.7%)	22 (0.7%)	
High	3027 (87.7%)	396 (11.9%)	18 (0.4%)	
Marital status				< 0.001
Never married	2484 (89.3%)	262 (10.1%)	15 (0.6%)	
Currently married	3958 (89.0%)	459 (10.6%)	17 (0.4%)	
Previously married	257 (71.8%)	87 (24.9%)	15 (3.3%)	
Region				0.35
Lima	3205 (88.3%)	392 (11.1%)	20 (0.6%)	
Arequipa	984 (87.7%)	122 (11.2%)	12 (1.1%)	
Callao	785 (90.5%)	74 (8.9%)	6 (0.6%)	
Cusco	469 (90.0%)	55 (9.8%)	1 (0.2%)	
Trujillo	927 (87.5%)	123 (12.0%)	6 (0.5%)	
Tumbes	329 (87.4%)	42 (12.1%)	2 (0.5%)	
Migrated alone				0.31
No	4798 (87.8%)	593 (11.6%)	37 (0.6%)	
Yes	1901 (89.9%)	215 (9.6%)	10 (0.5%)	
Refugee				0.70
No	6065 (88.5%)	721 (10.9%)	39 (0.6%)	
Yes	634 (87.7%)	87 (11.9%)	8 (0.4%)	
Worked last week				< 0.001
No	911 (82.6%)	177 (16.2%)	21 (1.2%)	
Yes	5788 (89.5%)	631 (10.0%)	26 (0.5%)	
Health insurance				0.15
No	6358 (88.6%)	745 (10.8%)	45 (0.6%)	
Yes	341 (85.6%)	63 (14.2%)	2 (0.2%)	

Percentages are in rows

On the other hand, 128 (2.0%; 95% CI 1.5–2.7%) subjects reported having a disability. Disability was more frequent among those aged ≥ 50 years (6.4% vs. 1.2% among those < 30 years; p < 0.001), those with lower education (p = 0.003), and among those who did not work during the previous week (p = 0.002; Table 3).

**Table 3:** Characteristics of the study population by disability status

	Disability status		p-value
	No	Yes	
	(n = 7426)	(n = 128)	
Sex			0.15
Female	3411 (97.7%)	68 (2.3%)	
Male	4015 (98.3%)	60 (1.7%)	
Age (in years)			< 0.001
< 30	3824 (98.8%)	34 (1.2%)	
30–39	2216 (98.0%)	34 (2.0%)	
40–49	900 (96.9%)	26 (3.1%)	
≥ 50	486 (93.6%)	34 (6.4%)	
Education level (in years)			0.003
< 7	732 (95.5%)	28 (4.5%)	
7–11	2333 (98.2%)	33 (1.8%)	
≥ 12	4361 (98.3%)	67 (1.7%)	
Socioeconomic status			0.08
Low	727 (98.2%)	9 (1.8%)	
Middle	3321 (98.6%)	56 (1.4%)	
High	3378 (96.9%)	63 (3.1%)	
Marital status			0.38
Never married	2710 (98.0%)	51 (2.0%)	
Currently married	4370 (98.1%)	64 (1.9%)	
Previously married	346 (96.6%)	13 (3.4%)	
Region			0.34
Lima	3538 (97.9%)	79 (2.1%)	
Arequipa	1110 (99.3%)	8 (0.7%)	
Callao	850 (98.4%)	15 (1.6%)	
Cusco	523 (99.6%)	2 (0.4%)	
Trujillo	1039 (98.4%)	17 (1.6%)	
Tumbes	366 (98.1%)	7 (1.8%)	
Migrated alone			0.48
No	5342 (98.1%)	86 (1.9%)	
Yes	2084 (97.8%)	42 (2.2%)	
Refugee			0.24
No	6710 (98.1%)	115 (1.9%)	
Yes	716 (96.7%)	13 (3.3%)	
Worked last week			0.002
No	1070 (96.3%)	39 (3.7%)	
Yes	6356 (98.3%)	89 (1.7%)	
Health insurance			0.61
No	7029 (98.0%)	119 (2.0%)	
Yes	397 (97.5%)	9 (2.5%)	

Percentages are in rows

### Migration, Multimorbidity and Disability

In multivariable model, after controlling by sex, age, education level, socio-economic status and region, neither, having migrated alone (PR = 0.92; 95% CI 0.74–1.15) nor being a refugee (PR = 0.98; 95% CI 0.67–1.44) were associated with multimorbidity. Similarly, having migrated alone (PR = 1.21; 95% CI 0.74–1.99) and having a refugee status (PR = 1.72; 95% CI 0.71–4.18) were not associated with disability.

## Discussion

### Main Findings

Our findings show a low prevalence of multimorbidity and disability among Venezuelan migrants living in Peru. Neither migration alone nor being a refugee were predictors of multimorbidity or disability. While multimorbidity may not be highly prevalent in this population, Venezuelan migrants do need adequate healthcare to control the—unique—chronic condition they may have as more than 10% of subjects presented at least one of the twelve chronic conditions evaluated and most of them were not taking treatment for these conditions.

### Explanations and Implications of Findings

Our findings suggest there is a low self-reported prevalence of common chronic conditions (e.g., asthma, hypertension, and type 2 diabetes) as well as of multimorbidity and disability in Venezuelan migrants in Peru. These findings could be explained by the profile of the migrants. Arguably, young individuals, those economically active, those with higher education, and those with better health were the first to migrate seeking better living standards. Our results suggest that currently multimorbidity could not be a top priority among these migrants; nevertheless, given the chronic nature of these conditions and the lack of treatment reported, some actions should be prioritized for preventive healthcare decision-making and allocation of resources in Peru. In addition, surveillance of this population should be guaranteed to appropriately detect the health needs of this population, including but not limited to anxiety and depressive symptoms as well as other mental health issues.

Prevalence estimates herein reported were lower than those observed in the Venezuelan Study of Cardiometabolic Health (EVESCAM), a national survey in Venezuela [19]. Differences reported between the EVESCAM and the ENPOVE may be related to the methodology used for each study. Whereas the ENPOVE was based on self-reported conditions, the EVESCAM was based on anthropometric and clinical markers (e.g., fasting glucose, blood pressure measurements, etc.). In addition to that, mean age in the ENPOVE was, on average, 10 years below than that in the EVESCAM, highlighting the younger age at migration with the probability of having low prevalence of chronic conditions. Another explanation, as pinpointed above, could be that healthier and wealthier individuals are more likely to migrate [20], especially at the crisis onset. This phenomenon may contribute to the well-known healthy migrant effect, which implies the observed morbidity and mortality advantage of migrants relative to the majority population is the host country. This hypothesis could have two relevant implications. First, currently chronic conditions may not carry a large burden among these migrants, but they do need to adequate care for acute diseases and other prophylactic care (e.g., vaccination or safe sex). Second, assuming these migrants will stay in Peru for long, primary prevention, and in some cases secondary prevention strategies, are needed to secure they age as healthy as possible. In so doing, we will avoid large health expenses in these migrants in the future.

### Public Health Implications

Usually, migrants are exposed to different health risks and inequities in access to healthcare [21]. Our findings suggest there is a very low proportion of individuals with any health insurance. This could be due to differences in need (i.e., few people with illnesses), preferences, lack of information and knowledge about the Peruvian health systems, formal barriers (e.g., fees, waiting times, etc.), and travel distances. Emphasis should be placed to guarantee appropriate access to healthcare among Venezuelan migrants, especially because the mental health and wellbeing of migrants and their families are relevant [22]. Since 2004, mental health has gained relevance in Peru, resulting in the promotion of a mental health reform within the national healthcare system [23]. However, in 2012, this reform materialized including a restructuring of mental health service delivery at the primary and secondary care levels and the introduction of supporting services to improve patient recovery and reintegration into society [23]. Other preventive strategies, including screening for hypertension and type 2 diabetes, may be a good approach to detect cases of these chronic conditions among Venezuelan migrants and reduce the potential impact on our health system. As pointed out by the UCL-Lancet Commission on Migration and Health [10], migrants have right to health and this should be guaranteed by making health coverage universal [24] and for instance, promoting access to quality essential healthcare services and access to effective, safe, quality and affordable essential medicines and vaccines [25].

Although migrating alone or as a refugee was not associated with multimorbidity and disability in this study, migration and adaptation to the new environment are usually stressful experiences and may further affect mental health, especially among those without social support or a solid network [26]. Securing good mental health in the migrant population and social care could also have implications for the nationals. A recent systematic review show that many of the existing interventions are focused on post-traumatic stress disorder and trauma-related symptoms, with less attention for depression and anxiety disorders. Impaired mental health could lead to violent acts affecting national individuals [27].

### Strength and Limitations

This study benefited from a large population-based survey to obtain appropriate estimates of the health profile of Venezuelan migrants living in Peru. However, this work has limitations that should be highlighted. First, selection bias may arise because specific cities were chosen for the study. Lima and Callao were selected because they concentrate the greatest proportion of Venezuelans in Peru. Tumbes, in northern Peru, was selected because it is the entry point at the border with Ecuador. Trujillo and Arequipa were selected because they are the second and third city concentrating Venezuelan migrants, and Cusco, a cosmopolitan city, was chosen because tourism generates work in different related activities. Even though our results are applicable to a large proportion of the Venezuelan population in Peru, we cannot rule out that other patterns may exist among Venezuelan migrants in other cities in Peru. Second, health and chronic conditions were not assessed objectively, but instead, these were self-reported. Unawareness could have biased our results towards lower prevalence estimates. In fact, population-based prevalence estimates of chronic conditions are much higher in Venezuela than herein reported [19]. Although awareness is close to 50% in the case of type 2 diabetes, this may not totally explain the lower prevalence results here estimated [28]. Our results thus call for further research with stronger ascertainment methods. Third, anxiety and depressive symptoms, key conditions to be assessed among displaced individuals, were not appropriately assessed, and included only by self-report. This highlights the need of a more adequate approach to evaluate mental health disorders in this group. Finally, the cross-sectional design of the ENPOVE survey only allows to study associations without causality claims.

## Conclusions

Low multimorbidity and disability prevalence was found among Venezuelan migrants living in Peru, suggesting there is an underlying healthy migrant effect. Migrating alone or being a refugee was not associated with multimorbidity or disability.
